# Anticonvulsant Activity of B2, an Adenosine Analog, on Chemical Convulsant-Induced Seizures

**DOI:** 10.1371/journal.pone.0067060

**Published:** 2013-06-25

**Authors:** Min Li, Ruixia Kang, Jiangong Shi, Gengtao Liu, Jianjun Zhang

**Affiliations:** 1 State Key Laboratory of Bioactive Substance and Function of Natural Medicines, Institute of Materia Medica, Chinese Academy of Medical Sciences and Peking Union Medical College, Beijing, China; 2 Department of Clinical Pharmacology, Beijing Hospital of the Ministry of Health, Beijing, China; University of Arizona, United States of America

## Abstract

Epilepsy is a chronic neurological disorder characterized by recurrent seizures. However, approximately one-third of epilepsy patients still suffer from uncontrolled seizures. Effective treatments for epilepsy are yet to be developed. *N* ^6^-(3-methoxyl-4-hydroxybenzyl) adenine riboside (B2) is a N^6^-substitued adenosine analog. Here we describe an investigation of the effects and mechanisms of B2 on chemical convulsant-induced seizures. Seizures were induced in mice by administration of 4-aminopyridine (4-AP), pentylenetetrazol (PTZ), picrotoxin, kainite acid (KA), or strychnine. B2 has a dose-related anticonvulsant effect in these chemical-induced seizure models. The protective effects of B2 include increased latency of seizure onset, decreased seizure occurrence, shorter seizure duration and reduced mortality rate. Radioligand binding and cAMP accumulation assays indicated that B2 might be a functional ligand for both adenosine A_1_ and A_2A_ receptors. Furthermore, DPCPX, a selective A_1_ receptor antagonist, but not SCH58261, a selective A_2A_ receptor antagonist, blocked the anticonvulsant effect of B2 on PTZ-induced seizure. c-Fos is a cellular marker for neuronal activity. Immunohistochemical and western blot analyses indicated that B2 significantly reversed PTZ-induced c-Fos expression in the hippocampus. Together, these results indicate that B2 has significant anticonvulsant effects. The anticonvulsant effects of B2 may be attributed to adenosine A_1_ receptor activation and reduced neuronal excitability in the hippocampus. These observations also support that the use of adenosine receptor agonist may be a promising approach for the treatment of epilepsy.

## Introduction

Epilepsy affects up to 1% of the population [1]. While many drugs have been developed for the treatment of epilepsy, approximately one-third of epilepsy patients still have uncontrolled seizures, with an even larger percent suffering from the side effects of antiepileptic drugs [2].

One emerging strategy in the search for new antiepileptic drugs has been to target the adenosine pathway. Adenosine is an endogenous anticonvulsant. It may influence the release of excitatory amino acids and other neurotransmitters or directly inhibit spontaneous neuronal firing and synaptic transmission [3]. However, cardiovascular side effects, low brain permeability, and a short time course of action may limit its application in epilepsy treatment. Given that the anticonvulsant activity of adenosine is primarily mediated though the adenosine A_1_ receptor [4], adenosine receptor agonists may hold promise for the development of epilepsy drugs [5].

The main approach for identifying adenosine receptor agonists has been to use adenosine analogs to investigate the structure-activity characteristics of adenosine at adenosine receptors [6]. Most of the analogs that have intrinsic activity are modified in the N^6^- or 2-position of the adenine moiety [7], [8]. Agonist selectivity for the A_1_ adenosine receptor is typically accomplished through a substitution at the N^6^-position of adenosine, such as N^6^-cyclopentyladenosine (CPA) [9]. However, N^6^-[2-(3,5-dimethoxyphenyl)-2-(2-methylphenyl)-ethyl]adenosine (DPMA), a potent agonist for the A_2A_ adenosine receptor, is also a N^6^-substituted adenosine derivative.

Modifying the N^6^-position of the adenine moiety enabled us to synthesize a series of adenosine analogs, which we examined in terms of binding properties and anticonvulsant action. Among hundreds of compounds, B2, an N^6^-substituted adenosine derivative, was found to have potential in the treatment of epilepsy. A previous study showed that B2 remarkably increased PC12 cell survival in serum deprivation-induced apoptosis and had a moderate affinity for rat adenosine A_2A_ receptors [10]. The present study aimed to generate a pharmacological profile for B2. We tested its anti-epileptic activity in different models of chemically induced seizures, its radioligand binding properties at the adenosine A_1_ and A_2A_ receptors, its modulatory effects on both adenosine A_1_ and A_2A_ receptors, and its effects on abnormally elevated neuronal excitability.

## Materials and Methods

### Animals

Adult male ICR mice (weighing 22±2 g) and rats (weighing 300±10 g) were obtained from Vital River Laboratories (Beijing, China). The animals were housed in acrylic cages (45×60×25 cm) with water and food available ad libitum in a sound-proof room (22±1°C) with an artificial 12-h light/dark cycle (light from 8∶00 a.m. to 8∶00 p.m.). To ensure adequate adaptation to their new environment, the mice were kept in the departmental holding room for one week prior to testing. Experimental procedures were in compliant with the guidelines set by the National Institute of Health and were approved by the Animal Care Committee of the Peking Union Medical College and the Chinese Academy of Medical Sciences. All efforts were made to minimize suffering.

### Drugs

**Figure 1:**
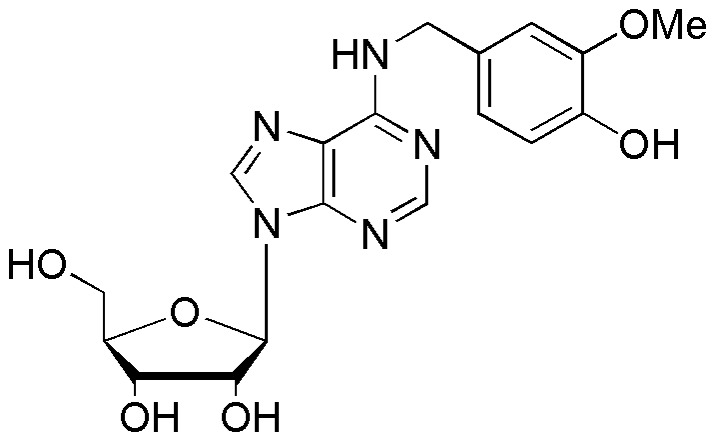
The chemical structure of B2 (*N* ^6^-(3-methoxyl-4-hydroxybenzyl) adenine riboside.

B2 (Fig. 1, purity >99%) was synthesized by Professor Jiangong Shi at the Department of Phytochemistry, Materia Medica Institute, Beijing, China. The structure of B2 was identified to be N^6^-(3-methoxyl-4-hydroxybenzyl) adenine riboside by nuclear magnetic resonance and mass spectrometry data analysis. The following drugs were also used: [^3^H] 8-cyclopentyl-1,3-dipropylxanthine ([^3^H]DPCPX, Radiolabeled Chemicals), [^3^H] 3-(3-hydroxypropyl)-7-methyl-8-(m-methoxystyryl)-1-propargylxanthine ([^3^H]MSX- 2, Radiolabeled Chemicals), rolipram (Sigma), cAMP (Sigma), [^3^H]cAMP (Perkin-Elmer), pentylenetetrazol (PTZ; Sigma), picrotoxin (Sigma), kainic acid (KA;Sigma), strychnine (Sigma), diazepam (China National Pharmaceutical Group Corporation), carbamazepine (Sigma), 8-Cyclopentyl-1,3-dipropylxanthine (DPCPX, Tocris), and 5-amino-7-(β-phenylethyl)-2-(2-furyl)-pyrazolo-[4,3-e]-1,2,4-triazolo[1,5-c]-pyrimidine (SCH 58261, Tocris). The rabbit polyclonal anti-c-Fos antibody was purchased from Santa Cruz Biotechnology, Inc. The VectaStain® Elite ABC kit and 3,3′-diamino-benzidine-tetrahydrochloride (DAB) were purchased from Vector Laboratories.

### Treatments

B2, diazepam, carbamazepine, picrotoxin, 4-AP, and strychnine were suspended in 1% Tween 80/physiological saline. KA was dissolved in saline and the pH was adjusted to neutrality. The final concentration of KA was 0.7 µg/µl, and the convulsant dose was 1 µl (i.c.v.). The convulsant doses and routes of administration for the other drugs were as follows: PTZ, 85 mg/kg (s.c.); picrotoxin,3.5 mg/kg (s.c.); 4-AP, 13 mg/kg (s.c.); and strychnine,1.5 mg/kg (i.p.) (all onvulsants caused seizure in 100% of animals). B2 (1.25, 5, 20 mg/kg), diazepam (10 mg/kg) or carbamazepine (80 mg/kg) was administered (i.p.) 15 min before administration of PTZ, picrotoxin, KA, 4-AP, or strychnine. The selective adenosine receptor antagonist DPCPX or SCH58261 was administered (i.p.) 5 min before administration of B2 or vehicle.

### Cell Culture and Membrane Preparation

#### Cell culture

Chinese hamster ovary (CHO) cells expressing recombinant human A_1_/A_2A_ receptors (provided by Professor R. B. Su of the Institute of Pharmacology and Toxicology, Academy of Military Medical Sciences, Beijing, China) were cultured in Dulbecco’s Modified Eagles Medium with the F12 nutrient mixture (DMEM/F12) containing no nucleosides, and with 10% fetal calf serum, penicillin (100 U/ml), streptomycin (100 µg/ml), L-glutamine (2 mM), and Geneticin at 37°C in a 5% CO_2_/95% atmosphere. The cells used to determine cAMP levels had viability >95%, as assessed by trypan blue exclusion.

#### Membrane preparation

Male Wistar rats were sacrificed. The cerebral cortex and striatum were then removed and homogenized in 10 volumes of ice-cold 50 mM Tris–HCl buffer (pH 7.4). The homogenates were centrifuged (48,000 ×g) at 4°C for 10 min, the supernatant decanted, and the pellet resuspended in the same volume of buffer before being centrifuged again. The final pellets were resuspended in 50 mM Tris–HCl buffer containing adenosine deaminase (1.2 U per mg of protein) to remove endogenous adenosine.

### Binding Assay at the A_1_ and A_2A_ Receptors

The binding affinity of B2 at adenosine A_1_ receptors was tested in rat cortical membranes using 0.2 nM [^3^H]DPCPX (85 Ci/mmol). Binding affinity at adenosine A_2A_ receptors was tested in rat striatal membranes using 0.75 nM [^3^H]MSX-2 (104.8 Ci/mmol). The binding assay was carried out in Tris–HCl buffer (50 mM, pH 7.4) as previously described [11], [12]. Briefly, assays were performed by first incubating the mixtures on a shaking water-bath at 25°C for 30 min. Termination of the incubation was performed by rapid filtration through GF/B glass fiber filters. The radioactivity on the filter was measured using a scintillation counter. Nonspecific binding was defined as binding activity in the presence of 10 µM of 2-chloroadenosine (2-CADO) or 50 µM of NECA for adenosine A_1_ and A_2A_ binding assays, respectively. For saturation analysis, the K_D_ value was set at 0.14 nM and the B_max_ value was 2290 fmol/mg protein for binding of [^3^H]DPCPX to the A_1_ adenosine receptor. Meanwhile, the K_D_ value was set at 11.48 nM and the B_max_ value was 5651 fmol/mg protein for binding of [^3^H]MSX-2 to the A_2A_ adenosine receptor. Inhibition curves were determined using eight different concentrations ranging from 10^−5^ to 10^−8 ^M. The inhibition constants (K_i_) were calculated using an equation from Cheng and Prusoff [13].

### Cyclic AMP Accumulation Assay

Intracellular cyclic adenosine monophosphate (cAMP) levels were measured using a competitive protein binding method [14]. To measure A_1_/A_2A_ receptor-mediated accumulation of cAMP, cells were grown as confluent monolayers in 24-well cluster dishes. The cells were washed twice with 2 mL of medium containing 20 mM HEPES that had been prewarmed to 37°C, pH 7.4. The cells were then preincubated for 15 min in 0.4 mL fresh medium containing 30 µM of the cAMP phosphodiesterase inhibitor rolipram. B2 was added in 0.1 mL of medium and incubations continued for 15 min. The reaction was then terminated by removing the supernatant, and cells were lysed with the addition of 500 µl of 0.1 M ice-cold HCl. The cell lysate was centrifuged (4,000×g) at 4°C for 10 min. The supernatants or cAMP standards (0–8 pmol) were incubated with [^3^H]cAMP (27 Ci/mmol) and cAMP binding protein in 96-well microtiter plates at 4°C for 150 min. Free and bound [^3^H]cAMP were separated by filtration with Whatman GF/B filters using a semi-automatic cell harvester. Each filter was rinsed with 3 mL of 50 mM Tris-HCl, pH 7.4. Bound radioactivity was measured by liquid scintillation spectrometry.

### KA-induced Seizures

The left lateral ventricle (0.6 mm posterior, 1.9 mm lateral, and 2.1 mm ventral to bregma) was cannulated as described [15]. After a 7-day recovery period, mice were injected with either B2 (1.25, 5, 20 mg/kg) or vehicle. Twenty minutes later, an intracerebroventricular injection of KA (0.7 µg/µl, 1 µl) was administered without anesthesia. The animals were then observed for a minimum of 60 min. Seizure severity was determined according to the scoring system described by Racine [16]. At the end of the experiment, cannulae placement was verified.

### Seizures Induced by Strychnine or 4-AP

Mice were injected with B2 (1.25, 5, 20 mg/kg), carbamazepine (80 mg/kg) or vehicle 20 min before administration of strychnine (1.5 mg/kg) or 4-AP (13 mg/kg). The animals were observed for at least 15 min after the administration of strychnine and 1 h after the administration of 4-AP. The time of latency to seizure onset and mortality rate were recorded.

### Seizure Induced by Picrotoxin or PTZ

Mice were injected with B2 (1.25, 5, 20 mg/kg), diazepam (10 mg/kg), or vehicle 20 min before the administration of picrotoxin (3.5 mg/kg) or PTZ (85 mg/kg). The mice were observed for a minimum of 1 h after administration of picrotoxin or 15 min after administration of PTZ. The latency of seizure onset, number of seizures, and mortality rate was recorded for the picrotoxin-injected mice. For the PTZ-injected mice, we recorded the latency of seizure onset, number of seizures, and presence of clonic seizures.

To evaluated the contribution of adenosine receptors in the anticonvulsant effect of B2, a selective A_1_ receptor antagonist DPCPX or a selective A_2A_ receptor antagonist SCH58261 or their vehicles was administered to the mice 5 min before B2 (5 mg/kg) or vehicle treatments. Twenty min later, mice were admdinistered with PTZ (85 mg/kg). Then the mice were observed for 15 min and the latency of seizure onset was recorded.

### General Procedure for c-Fos Assay

Mice were injected with either B2 (5 mg/kg) or vehicle 20 min before administration of PTZ (85 mg/kg). Two hours later, mice were either anesthetized for c-Fos analysis using immunohistochemistry or decapitated or western blotting.

### C-Fos Analysis by Immunohistochemistry

Mice were anesthetized with chloral hydrate (400 mg/kg, i.p.) and perfused through the heart with saline solution followed by ice-cold 4% paraformaldehyde (PFA) in 0.1 M phosphate buffer (pH 7.4). Their brains were then removed, post-fixed in 4% PFA for 24 h at 4°C, and immersed in 30% sucrose at 4°C until they sank. Thereafter, coronal sections (30 µm) were cut using a freezing microtome (Leica RM2125).

Sections were immunostained using previously described methods [17]. Sections were first immersed in 0.3% Triton X-100 for 10 min at room temperature, and then in 3% hydrogen peroxide for 10 min to quench endogenous peroxidase activity. Sections were then rinsed twice in 10 mM phosphate-buffered saline (pH 7.2) containing 0.3% Triton X-100. This was followed by incubation with diluted normal blocking serum (Vector Elite ABC kit) for 30 min and then 1∶800 rabbit polyclonal anti-c-Fos (primary) antibodies overnight at 4°C. Biotinylated secondary antibody solutions and VectaStain® Elite ABC reagents were applied. The peroxidase reaction was visualized using DAB with nickel ammonium sulfate for black staining. Sections were mounted, dehydrated and cover slipped. As controls, adjacent sections were incubated without the primary antibody to confirm the absence of non-specific staining.

Using light microscopy (Nikon Eclipse 80i), c-Fos positive neurons in the hippocampus were identified by dense black nuclear staining. Locations in the brain were confirmed using the delineations in the Paxinos Atlas.

### C-Fos Analysis by Western Blot

Mice were decapitated and the hippocampus was quickly dissected on ice. The hippocampus was then homogenized in 20 volumes of cell lysis buffer. The homogenates were incubated on ice for 15 min and centrifuged at 12000×g for 5 min at 4°C. The supernatant containing the crude nuclear extract was collected and the protein content estimated using a Bradford assay. Protein samples (g) were electrophoresed in 10% SDS-PAGE at 150 V for 3 h. The samples were then electroblotted onto nitrocellulose membranes at 20 V. The membranes were blocked using 5% dry non-fat milk in 0.1% TBST solution, and incubated overnight on a shaker with anti-c-Fos rabbit antibody (SC-52g) diluted 1∶800 (Santa Cruz Biotechnology). On the following day, the blots were washed using 0.1% TBST solution and incubated for 2 h with anti-rabbit peroxidase-conjugated secondary antibody. The blots were then washed using 0.1% TBST solution. After adding super ECL plus solution, the protein bands were detected using ECL (Fuji Digital Science), as described by the manufacturer. The optical density of the intensity of the bands was measured using Gel Pro Analyzer (version 4.0).

### Statistical Analysis

The results were expressed as mean±S.E.M. Data were analyzed by t-test or one-way analysis of variance (ANOVA) followed by the Newman Keuls post-test for multiple comparisons. Fisher’s exact test was used to compare the rate of mortality between the controls and each of the other groups. For western blot analysis, the Student’s t-test was used to compare c-Fos expression between each group. Differences were considered statistically significant at *P*<0.05.

## Results

### Effect of B2 on KA-induced Seizures

**Figure 2:**
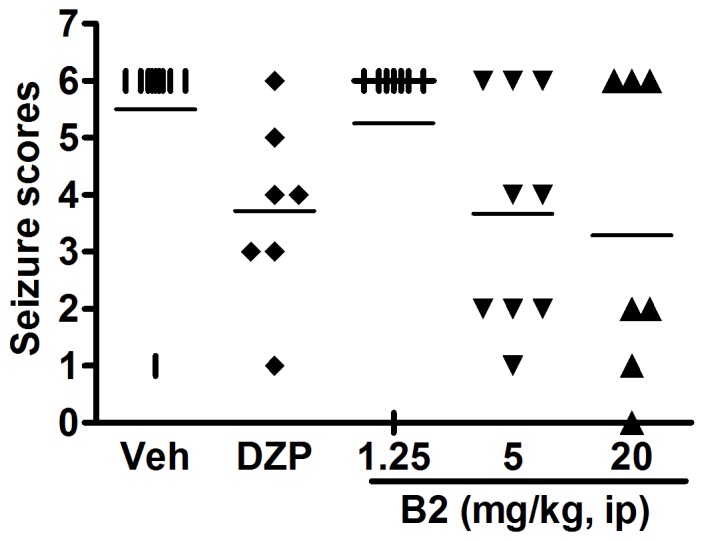
Anticonvulsant effects of B2 on KA-induced seizures in mice. The seizure score was recorded for each treatment group.

Intracerebroventricular administration of KA (0.7 µg) caused seizures in all control mice. The most severe seizures reached Stage 6, and were observed in eight of the nine mice. One mouse had Stage 1 seizure (*P*<0.05). Diazepam (10 mg/kg), the positive control, provided significant protection against KA-induced seizures in mice. B2 also had an anticonvulsant effect on the KA-induced seizures. The average seizure duration were 5.3±0.8, 3.7±0.7, and 3.3±1.0 min with 1.25, 5, and 20 mg/kg of B2, respectively (Fig. 2).

### Effects of B2 on Strychnine-induced Seizures

**Figure 3:**
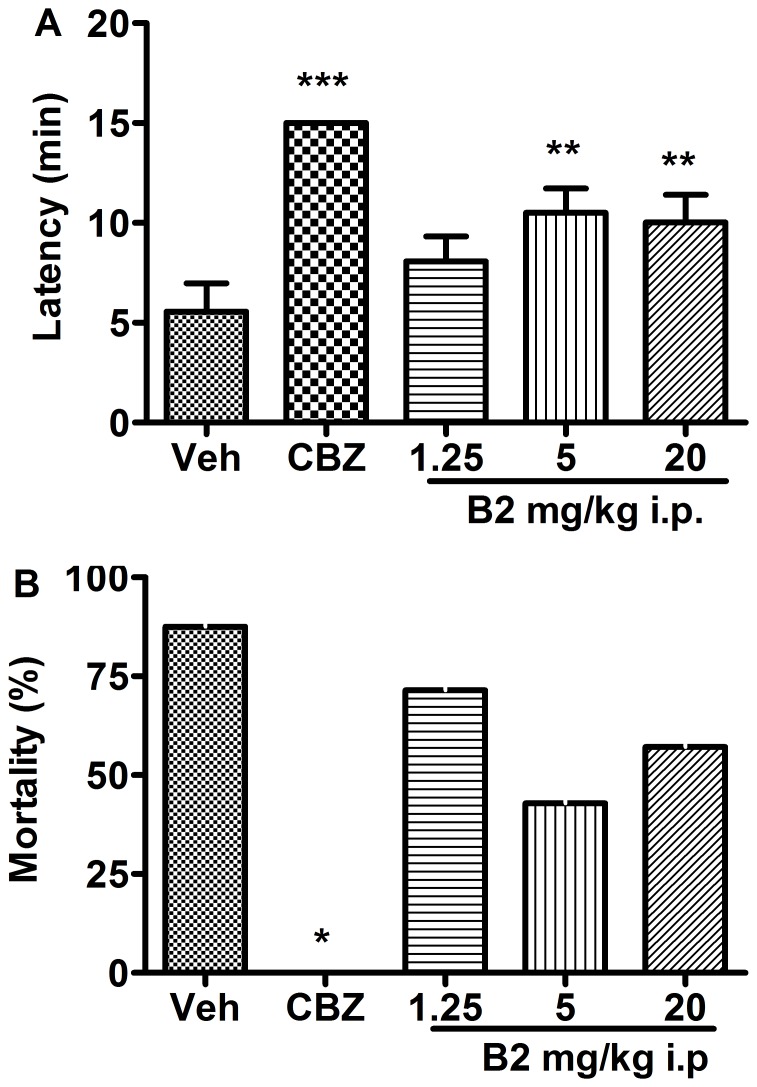
Anticonvulsant effects of B2 on strychnine-induced seizures in mice. (A) The latency of seizure onset was measured. Each column represents the mean ± S.E.M. (n = 7–8). ^*^ *P*<0.05, ^**^ *P*<0.01 and ^***^ *P*<0.001, compared with the control group (one-way ANOVA followed by Newman Keuls post-test). (B) The mortality rate in each treatment group is shown as a percentage. ^*^ *P*<0.05 compared with the control group (Fisher’s exact test).

Intraperitoneal administration of strychnine (1.5 mg/kg) caused seizures in all control mice. The mean latency to seizure onset was 5.6±1.4 min and the mortality rate was 88%. Carbamazepine (80 mg/kg), the positive control, provided complete protection against strychnine-induced seizures in mice. B2 had a significant effect on strychnine-induced seizures. The latency to seizure onset was prolonged by 45%, 88%, and 89% in the 1.25, 5, and 20 mg/kg groups, respectively (*F*(4,28)  = 3.01; *P*<0.05). The mortality rate was reduced to 71%, 43% and 57% in the 1.25, 5 and 20 mg/kg groups, respectively (Fig. 3).

### Effect of B2 on 4-AP Induced Seizures

**Figure 4:**
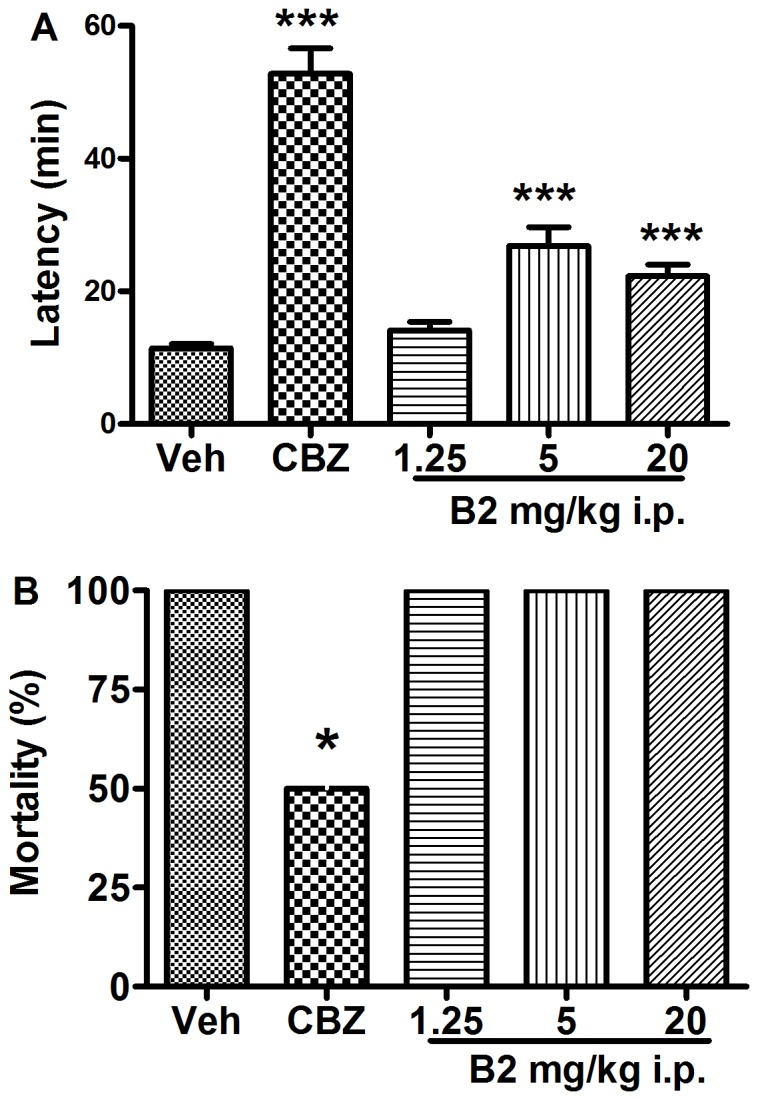
Anticonvulsant effects of B2 on 4-AP-induced seizures in mice. (A) The latency of seizure onset was measured. Each column represents the mean ± S.E.M. (n = 6–7). ^***^ *P*<0.001 compared with the control group (one-way ANOVA followed by Newman Keuls post-test). (B) The mortality rate in each treatment group is shown as a percentage.^ *^ *P*<0.05 compared with the control group (Fisher’s exact test).

Intraperitoneal administration of 4-AP (13 mg/kg) caused seizures in all control mice. The mean latency to seizure onset was 11.4±0.7 min and the mortality rate was 100%. Carbamazepine (80 mg/kg), the positive control, significantly prolonged the latency to seizure onset (*P*<0.001) and reduced the mortality rate (*P*<0.05). B2 significantly prolonged the latency to seizure onset in this model. The latency of seizure onset was prolonged by 25%, 135%, and 96% in the 1.25, 5, and 20 mg/kg groups, respectively (*F*(4,24) = 14.98; *P*<0.001) (Fig. 4). However, B2 did not have a significant effect on mortality rate.

### Effect of B2 on Picrotoxin-induced Seizures

**Figure 5:**
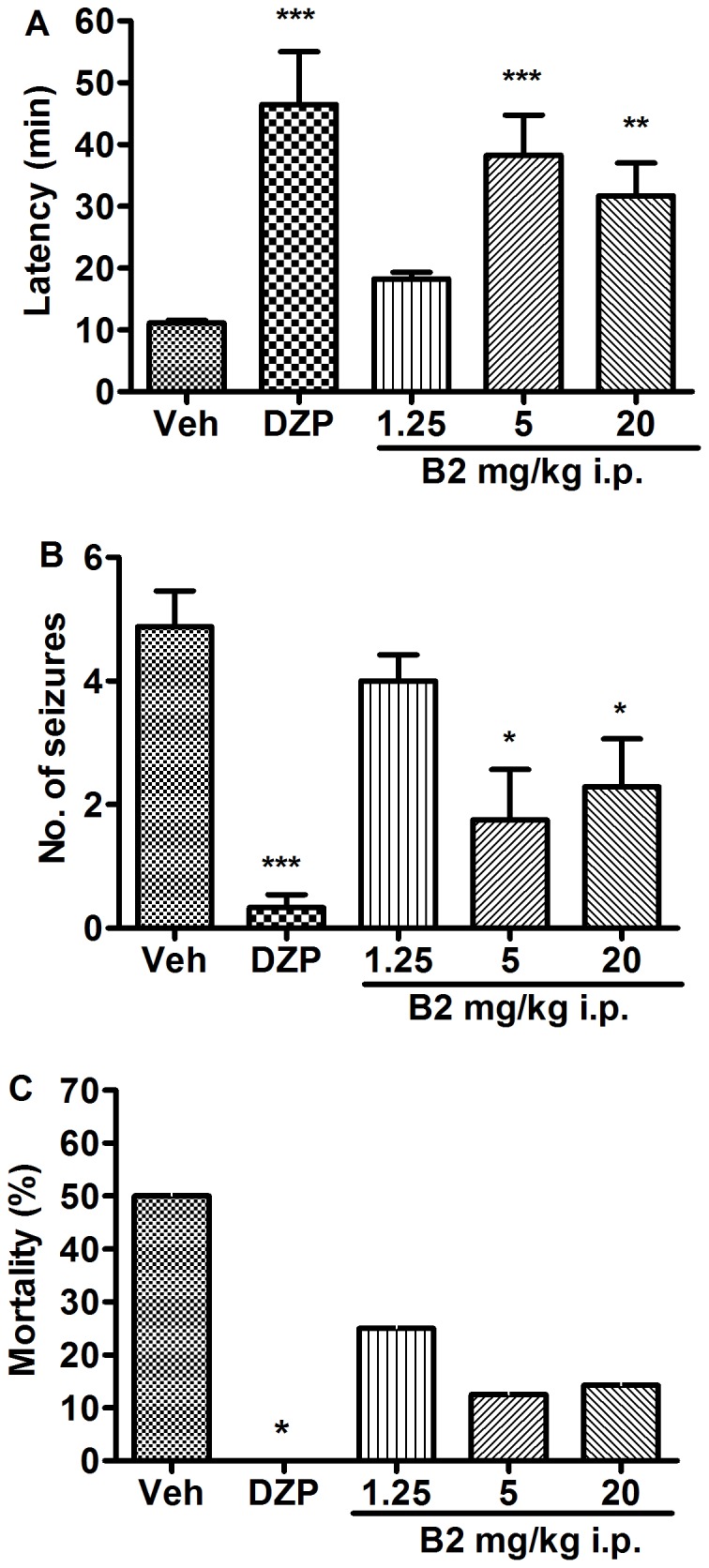
Anticonvulsant effects of B2 on picrotoxin-induced seizures in mice. (A) The latency of seizure onset and (B) the number of seizures were measured. Each column represents the mean±S.E.M. (n = 6–8). *P<0.05, **P<0.01 and ***P<0.001 compared with the control group (one-way ANOVA followed by Newman Keuls post-test). (C) The mortality rate in each treatment group is shown as a percentage. *P<0.05 compared with the control group (Fisher’s exact test).

Intraperitoneal administration of picrotoxin (3.5 mg/kg) caused seizures in all control mice. The mean latency to seizure onset was 11.2±0.4 min, the average number of seizures was 4.9±0.6, and the mortality rate was 50%. Diazepam significantly protected against picrotoxin-induced seizures in mice (*P*<0.001). B2 also had a significant effect on picrotoxin-induced seizures. The latency to seizure onset was prolonged by 64%, 242%, and 183% in the 1.25, 5, and 20 mg/kg groups, respectively (*F*(4,30) = 9.06, *P*<0.001). The number of seizures was reduced by 18%, 64%, and 53% in the 1.25, 5, and 20 mg/kg groups, respectively (*F*(4,30) = 4.92, *P*<0.01). The mortality rate was reduced to 25%, 13% and 25% in the 1.25, 5 and 20 mg/kg groups, respectively (Fig. 5).

### Effects of B2 on PTZ-induced Seizures

**Figure 6:**
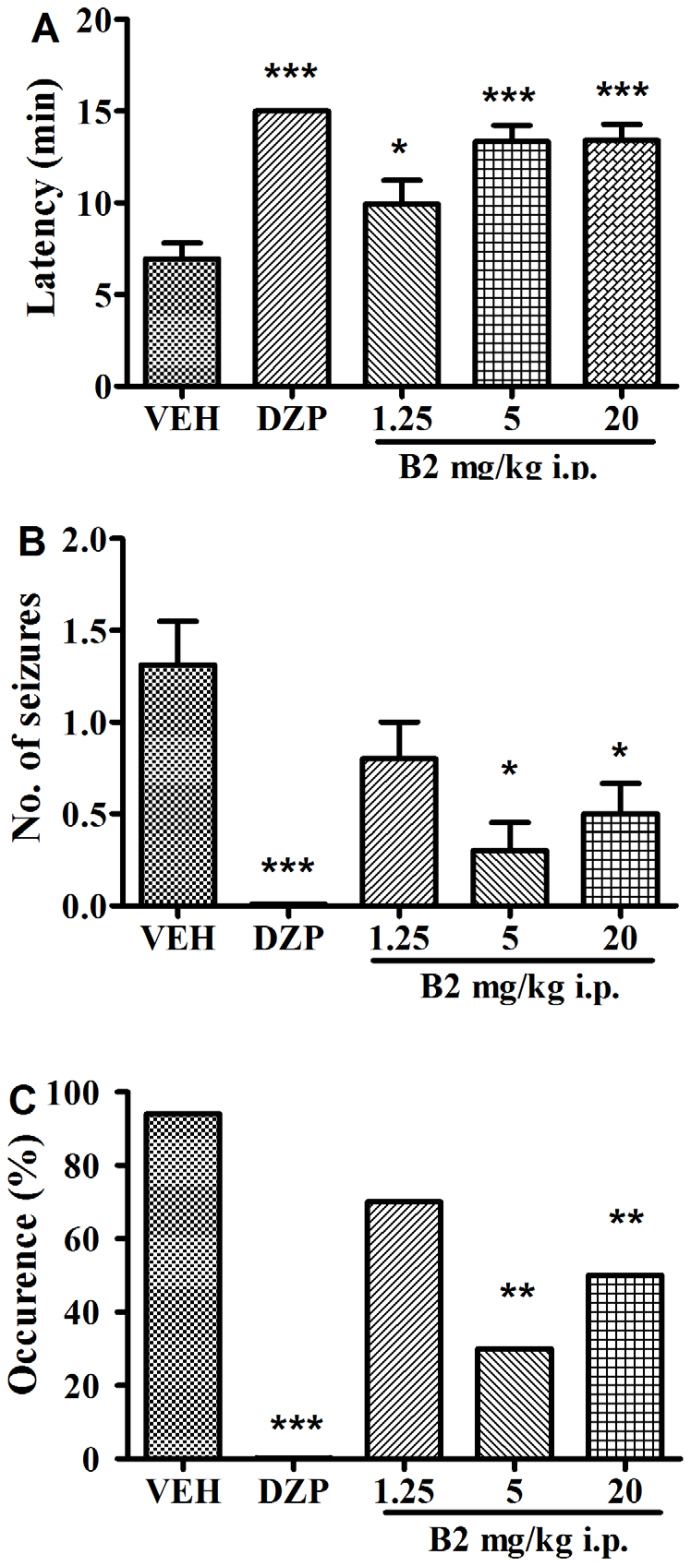
Anticonvulsant activity of B2 on PTZ-induced seizure in mice. (A) The latency of seizure onset, (B) the number of seizures and (C) the seizure occurrence were measured. Each column represents the mean ± S.E.M. (n = 8–16). ^*^ *P*<0.05, ^**^ *P*<0.01 and ^***^ *P*<0.001 compared with the control group (one-way ANOVA followed by Newman Keuls post-test).

Subcutaneous administration of PTZ (85 mg/kg) caused seizures in all control mice. The mean latency to seizure onset was 7.0±0.9 min, the average number of seizures was 1.3±0.2, and the mortality rate was 50%. Diazepam (10 mg/kg) completely protected against PTZ-induced seizures in mice (*P*<0.001). B2 had a significant effect on PTZ-induced seizures. The latency to seizure onset was prolonged by 43%, 89%, and 93% in the 1.25, 5, and 20 mg/kg groups, respectively (*F*(4,43) = 10.68; *P*<0.001). The number of seizures was reduced by 39%, 70% and 62% in the 1.25, 5 and 20 mg/kg groups, respectively (*F*(4,43) = 4.92; *P*<0.05). The seizure occurrence was reduced to 70%, 30% and 50% in the 1.25, 5 and 20 mg/kg groups, respectively. (Fig. 6).

### Radioligand Binding of B2 at Adenosine A_1_ and A_2A_ Receptor

**Figure 7:**
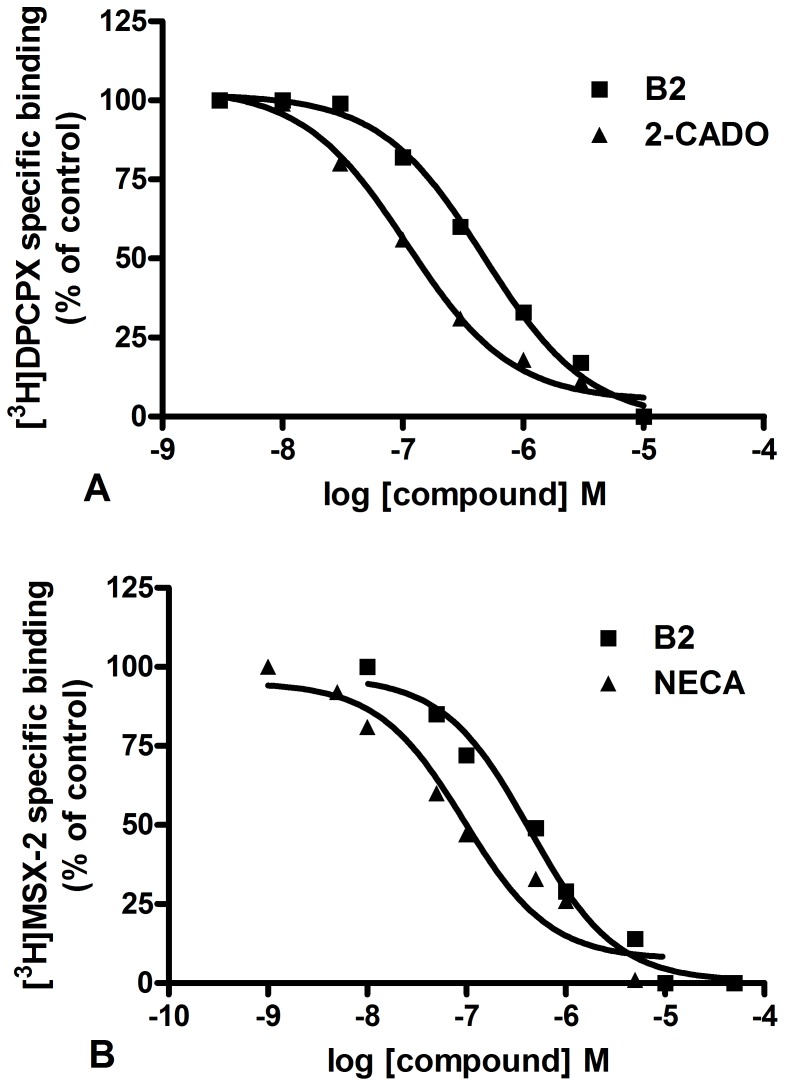
The competitive curves of the binding assay for B2 to the A_1_ and A_2A_ adenosine receptor. (A) Binding of B2 to the A_1_ adenosine receptor. (B) Binding of B2 to the A_2A_ adenosine receptor.

B2 was tested for its ability to displace [^3^H]DPCPX from the A_1_ adenosine receptor in rat cortical membranes and [^3^H]MSX-2 from the A_2A_ adenosine receptor in rat striatal membranes [10]. The adenosine A_1_ and A_2A_ receptor binding affinities for B2 were expressed as K_i_ values. In the A_1_ receptor binding assay, B2 had a K_i_ value of 0.42 µM (Fig. 7A), whereas NECA had a Ki value of 2.2 µM. In the A_2A_ receptor binding assay, B2 had a K_i_ value of 0.37 µM, whereas 2-CADO had a K_i_ value of 0.16 µM (Fig. 7B). These data indicated that B2 bound to the adenosine A_1_ and A_2A_ receptor with moderate affinity.

### Effect of B2 on cAMP Accumulation

**Figure 8:**
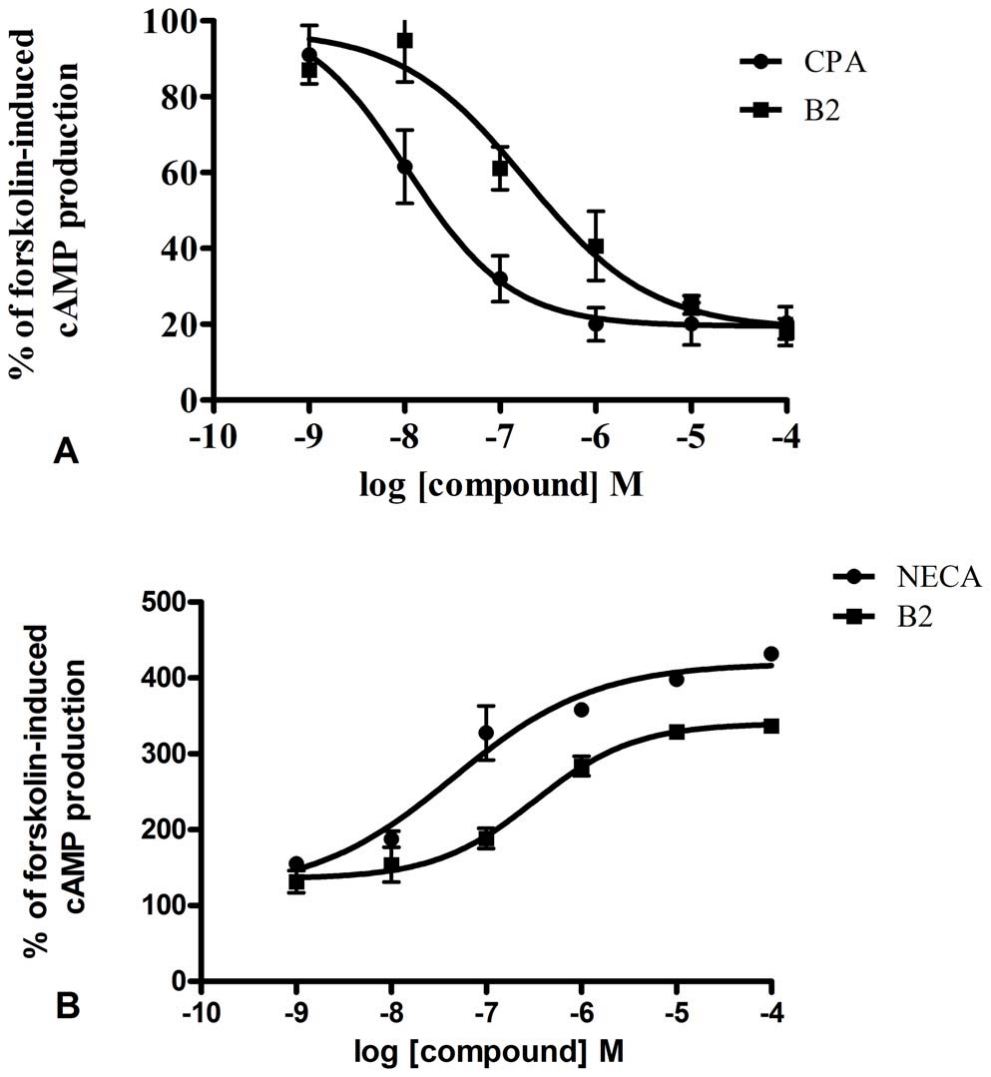
Dose response curves for a cAMP assay of B2. (A) Inhibition of forskolin-stimulated cAMP production by B2 or CPA in CHO cells expressing the human adenosine A_1_ receptor was fitted to a sigmoidal curve. (B) The accumulation of forskolin-stimulated cAMP by B2 or NECA in CHO cells expressing human adenosine A_2A_ receptor.

The modulatory effects of B2 on the adenosine A_1_ and A_2A_ receptors were evaluated by measuring cAMP accumulation in CHO cell lines that were stably transfected with recombinant human adenosine A_1_ or A_2A_ receptors. Activation of the A_1_ receptor inhibits adenylyl cyclase activity and subsequently decreases cAMP levels whereas activation of A_2A_ receptor produces the opposite effects [18], [19], [20]. Our results showed that B2 decreased the cellular cAMP level in the CHO cells stably expressing the A_1_ receptor with an EC_50_ value of 0.18 µM (Fig. 8A). On the other hand, B2 elevated cAMP levels in CHO cells expressing A_2A_ receptors with an EC_50_ value of 0.32 µM (Fig. 8B). These data indicate that B2 is a functional ligand for both adenosine A_1_ and A_2A_ receptor.

### Effect of DPCPX and SCH58261 on the Anticonvulsant Effect of B2

B2, at the dose of 5mg/kg, significantly prolonged the latency to seizure onset (*P*<0.05).

**Figure 9:**
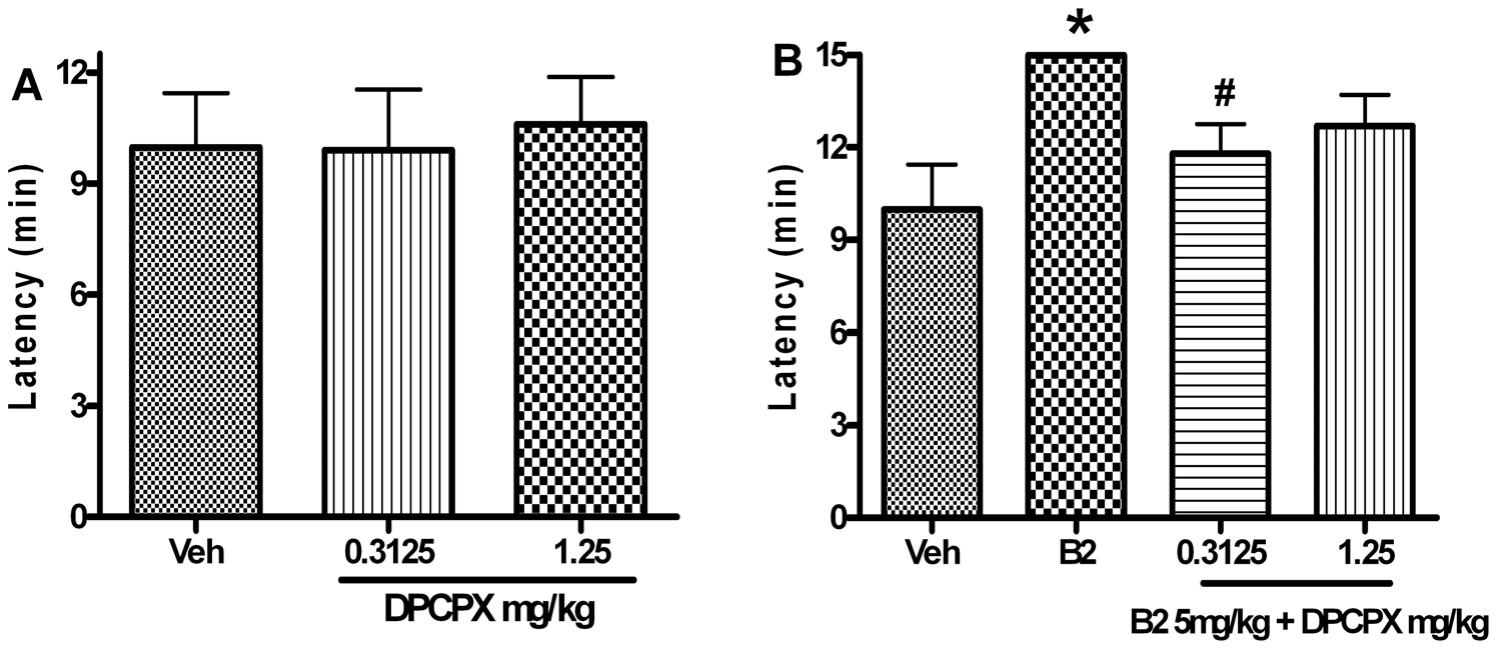
Antagonistic effects of DPCPX on the anticonvulsant activity of B2. Each column represents the mean ± S.E.M. (n = 9). ^*^ *P*<0.05 compared with the control group (Student’s *t*-test), ^#^ *P*<0.05 compared with the B2 group (Newman Keuls post-test).

DPCPX (0.3125–1.25 mg/kg) alone did not affect the onset time of seizure [*F*(2, 26) = 0.067, *P*>0.05] (Fig. 9A). However, DPCPX, at the ineffective dose on the seizure onset time, significantly blocked the delaying effect of B2 (5 mg/kg) on the onset times of seizure [F(2,26) = 4.156, *P*<0.05] (Fig. 9B).

**Figure 10:**
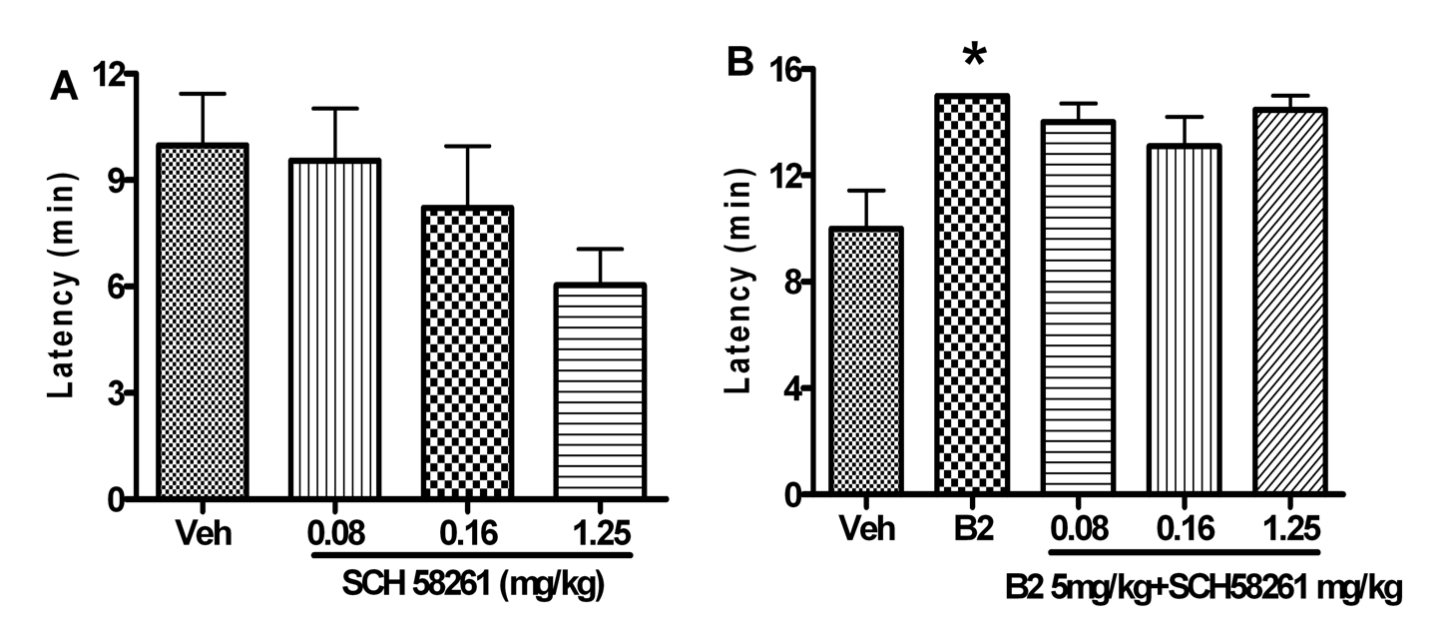
SCH58261 pretreatment had no effect on the anticonvulsant activity of B2. Each column represents the mean ± S.E.M. (n = 8–9). ^*^ *P*<0.05 compared with the control group (Student’s *t*-test), ^#^ *P*<0.05 compared with the B2 group (Newman Keuls post-test).

SCH58261 (0.08–1.25 mg/kg) alone did not affect the onset time of seizure [*F*(3,34) = 1.581, *P*>0.05] (Fig. 10A). Meanwhile, SCH58261 pretreatment did not affect the delaying effect of B2 on the seizure onset time [*F*(3,34) = 1.352, *P*>0.05], either (Fig. 10B).

### Effect of B2 on PTZ-induced c-Fos Expression using Immunohistochemistry Assay

**Figure 11:**
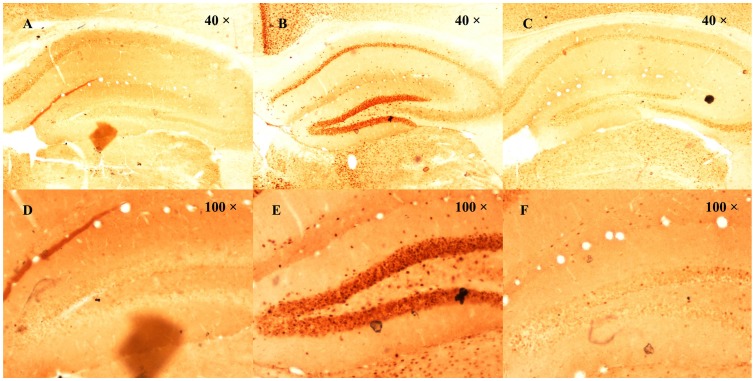
Effect of B2 on PTZ-induced c-Fos expression in the hippocampus. (1) Representative hippocampal c-Fos immunohistochemistry obtained from vehicle+vehicle (A), vehicle+PTZ (B), and B2+PTZ (C) treated mice. (2) Representative dentate gyrus c-Fos immunohistochemistry obtained from vehicle+vehicle (D), vehicle+PTZ (E), and B2+PTZ (F) treated mice.

To study the effects of B2 on neuronal excitability, we assessed c-Fos expression in the PTZ-induced seizure model. Fig. 11 shows representative photomicrographs of mice treated with vehicle+vehicle, vehicle+PTZ, or B2+PTZ. c-Fos positive neurons are shown with brown nuclear staining. After PTZ treatment, c-Fos expression showed a large increase in the dentate gyrus, a moderate increase in the CA1 region, and a small increase in the CA2/3 region. B2 significantly reversed the increase in the dentate gyrus and in CA1, and had a minor effect in the CA2/3 region. (Fig. 11).

### Effect of B2 on PTZ-induced c-Fos Expression using Western Blot Assay

**Figure 12:**
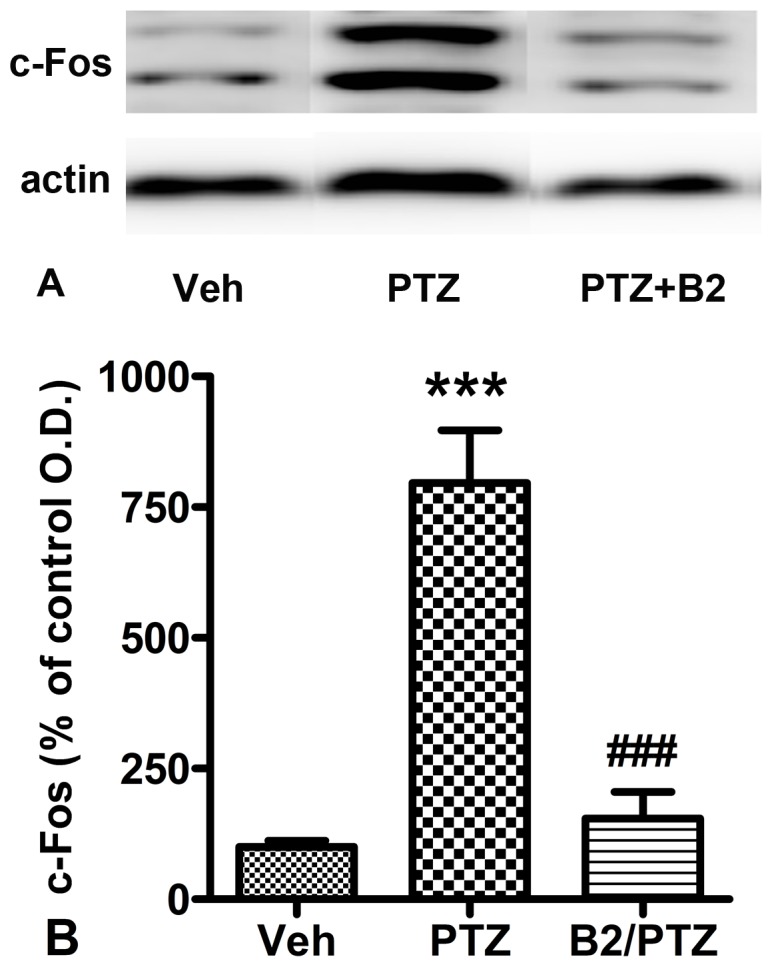
Effect of B2 on PTZ-induced c-Fos expression in the hippocampus. (A) Representative hippocampal c-Fos expression obtained from vehicle+vehicle, vehicle+PTZ, and B2+PTZ treated mice. (B) Quantitative analysis of c-Fos expression in the hippocampus using western blot analysis. Values are expressed as the mean±S.E.M. (n = 4). ^***^ *P*<0.001, compared with the control group. ^###^ *P*<0.001, compared with the PTZ group (Student’s t-test).

Western blot data indicated that PTZ significantly increased c-Fos expression in the hippocampus, where it was 8 times larger (*P*<0.001) than c-Fos expression in the vehicle control group (Fig. 12). B2 significantly reversed the PTZ-induced increase in c-Fos expression in the hippocampus, resulting in c-Fos expression that was only 1.5 times larger than in the vehicle control group.

## Discussion

The present study demonstrates that B2, an adenosine A_1_ and A_2A_ receptor agonist, has anticonvulsant effects against acute seizures triggered by a glutamate receptor agonist (KA), a glycine receptor antagonist (strychnine), a potassium channel blocker (4-AP) and γ-aminobutyric acid receptor(GABA) antagonists (PTZ and picrotoxin).

Chemical seizure models have been widely used in the preclinical evaluation of anticonvulsant properties of new drugs. For instance, the PTZ test represents a valid model for human generalized myoclonic seizures [21], strychnine is a chemoconvulsant model for primary generalized seizures, and 4-AP elicits acute seizures with generalized tonic-clonic features. Temporal lobe epilepsy and complex partial seizures are the most common forms of epilepsy in adults and are also the most difficult to treat [22]. KA-induced limbic seizures are considered to be a suitable model for temporal lobe epilepsy and complex partial seizures [23]. In this study, B2 had protective effects in all the above-mentioned models of epilepsy. That B2 was efficacious in all of these models implies potential pharmaceutical applications for a broad spectrum of seizure types.

Of the parameters measured, the latency to seizure onset seemed to be most sensitive to the anticonvulsant effects of B2. For instance, 1.25–20 mg/kg of B2 had an impact on the latency to seizure onset in all the models tested. In the case of 4-AP-induced seizures, B2 had little effect on the other parameters, but significantly prolonged the latency to seizure onset. This suggests that the most potent effect of B2 is to slow or block the synchronization or speed of spreading focal epileptiform activity.

The crucial role of adenosinergic neuromodulation in the control of seizure activity has been well established. Levels of endogenous adenosine may increase during epileptic seizures as a defense mechanism that acts to terminate ongoing seizure activity [24]. The A_1_ receptor is primarily responsible for the central effects of adenosine. The protective effect of adenosine A_1_ receptor stimulation against chemically induced seizures has also been well documented [25]. Previously tested chemicals models include PTZ, N-methyl-D-aspartate (NMDA), and bicuculline-methiodide. In addition, A_1_ receptor knockout mice experience spontaneous hippocampal seizures [26] and are hypersensitive to status epilepticus-induced brain injury [27]. Because B2 is an adenosine A_1_ receptor agonist, activation of the adenosine A_1_ receptor may underlie the anticonvulsant effects of B2 in chemically induced seizure models. In addition, the selective A_1_ receptor antagonist, DPCPX, but not A_2A_ antagonist, SCH58261, pretreatment blocked the anticonvulsant effects of B2 on the seizures induced by PTZ. This observation demonstrated that adenosine A_1_ receptors may have a more significant role in the anticonvulsant effects of B2 on PTZ-induced seizure.In contrast to the many studies on the adenosine A_1_ receptor, the potential of A_2A_ receptor activation for seizure suppression has been investigated only in a few studies and the results are controversial. Both anti-convulsive [28] and pro-convulsive [29] activity mediated by adenosine A_2A_ receptor activation have been documented. The reason for these discrepant findings may include differences in species, epilepsy models or doses. In addition, the selective A_2A_ receptor antagonist, SCH58261, pretreatment has no effect on the anticonvulsant effects of B2. The role of A_2A_ adenosine receptor activation in the antiepileptic activity of B2 remains to be clarified. More clarification of the relationship between adenosinergic receptors and B2 by further studies such as antagonistic tests in different seizure models maybe important and should be done in the future.

De Sarro et al [30] demonstrated that concomitant administration of low doses of adenosine A_1_ and A_2A_ receptor agonists, not active on their own, exerts a protective effect in seizure model, suggesting some interaction between the two types of adenosine receptors. Since B2 is a non-selective agonist for both A_1_ and A_2A_ adenosine receptors, synergistic mechanism may underlie the antiepileptic effect of B2. In addition, B2 has moderate affinity and efficacy at both A_1_ and A_2A_ adenosine receptor. Potent agonists for A_1_ or A_2A_ adenosine receptors could potentially be therapeutic agents for a variety of disorders, while moderate agonists may have advantages over potent agonists as a result of an increased selectivity of action. Such increased selectivity of action may provide the desired pharmacological effect with less toxicity.One third of epilepsy patients with refractory epilepsy are resistant to traditional AEDs because of drug-induced over-expression of multidrug transporters such as P-glycoprotein [31], [32]. Therefore, new therapeutic strategies, that take advantage of endogenous antiepileptic mechanisms of the brain, are less likely to be diminished by the activity of multidrug transporters. For that reason, in the search for new therapeutic approaches for drug-resistant focal epilepsies, the use of adenosine as an endogenous inhibitory neuromodulator appears to be promising [33]. Due to this emerging implication of adenosine in seizure management, a new field of research on adenosine-based therapies has been introduced including adenosine itself. Tested agents include adenosine receptor agonists and antagonists and adenosine kinase inhibitors [34]. In a wide range of experimental paradigms, pharmacological activation of adenosine A_1_ or A_2A_ receptors reduced convulsions induced by a variety of chemical and electrical stimuli [35], [36], [37]. More importantly, systemic application of the adenosine A_1_ receptor agonist 2-chloro-N^6^-cyclopentyladenosine (CCPA) suppressed epileptic discharges in a mouse model of drug-resistant epilepsy [38]. To bypass the profound peripheral effects (mainly cardiovascular), new approaches have been evaluated such as focal drug delivery [39] or the systemic co-application of a brain-permeable adenosine-receptor agonist with a non-brain-permeable adenosine-receptor antagonist [35], [37]. In addition, a recent study showed that the antidepressant drug tianeptine produced antiepileptic activity via adenosine A_1_ receptors, which may be useful for the treatment of epileptic patients with depression [40]. Clinical and basic research also supports that a ketogenic diet, which can specifically increase the influence of endogenous adenosine, offers benefits for epilepsy [41].

c-Fos labeling has been used extensively to evaluate neuronal activation following chemically induced seizures [42], [43], [44]. In our study, PTZ induced significant changes in Fos-immunoreactive cells in the hippocampus. Maximal activation occurred in the dentate gyrus, which is in agreement with previous reports [45], [46]. B2 significantly prevented the PTZ-induced increase in c-Fos expression in the dentate gyrus. The dentate gyrus is considered to act as a gate that prevents excessive activity from entering other hippocampal regions. Breakdown of this gate could be a critical event in the development of seizure activity [47]. The suppression of c-Fos expression in the dentate gyrus may be involved in the anticonvulsive effects of B2.

The optimal dose of B2 for rodent models of epilepsy seems to be 5 mg/kg, as the 1.25 and 20 mg/kg doses were less effective. The B2 dose-response relationship has an inverted U-shape, which indicates that, in other doses, B2 may have the opposite effect. However, the exact mechanisms underlying the U-shaped B2 dose-response relationship have yet to be clarified.

In conclusion, B2 is an adenosine analog that activates adenosine receptors, reduces neuronal excitability, and subsequently suppresses seizures induced by chemical convulsants. Therefore, B2 is a promising candidate for further preclinical studies aimed at the treatment of epilepsy. Further explorations of the potential antiepileptic mechanisms of B2 is necessary to enable the safe use of this compound in the treatment of epilepsy.
